# Permanent Spacers Are a Reliable Solution for Peri-prosthetic Shoulder Infection: A Systematic Review

**DOI:** 10.1007/s11420-020-09755-7

**Published:** 2020-04-08

**Authors:** Mattia Alessio-Mazzola, Ilaria Repetto, Antonio Russo, Antonio Clemente, Niccolò Ventura, Matteo Formica, Giorgio Burastero, Lamberto Felli

**Affiliations:** 1 Department of Surgical Sciences and Integrated Diagnostic (DISC), University of Genoa, Viale Benedetto XV, 6, 2° piano, 16132 Genoa, Italy; 2 Orthopedic Clinic, Ospedale Policlinico San Martino, Genoa, Italy; 3 Joint Replacement Unit, Azienda Ospedaliera Santa Corona, Pietra Ligure, Italy

**Keywords:** shoulder, infection, spacer, peri-prosthetic infection, articular shoulder spacer

## Abstract

**Background:**

Peri-prosthetic shoulder infection (PSI), a highly disabling complication of shoulder arthroplasty, often requires additional surgery and prolonged antibiotic therapy. Of strategies proposed to manage this devastating condition, the use of cement spacers, perhaps even as a definitive treatment, is debated.

**Questions/Purposes:**

We sought to systematically review the literature on antibiotic-loaded cement spacers as a viable, perhaps definitive, treatment for PSI, evaluating the eradication rates, mechanical reliability, and functional results related to its use.

**Methods:**

We conducted a systematic review of studies published from January 1, 1980, through September 1, 2019. Following the *Cochrane Handbook of Systematic Reviews of Interventions* and Preferred Reporting Items for Systematic Review and Meta-analysis, we searched for studies reporting functional and clinical outcomes in patients with PSI treated with a permanent spacer of the shoulder. Two independent reviewers searched eight databases, as well as reference lists of the retrieved articles.

**Results:**

After exclusion criteria were applied, 12 studies were included, involving a total of 143 patients. The mean age was 65.8 years; the mean follow-up was 37.4 months. A total of 133 patients (93%) were free from infection at latest follow-up. The mean post-operative active elevation of the shoulder ranged from 48.6 to 90°, the mean abduction ranged from 51 to 75°, and external rotation ranged from 3.6 to 29°. The mean Constant–Murley score ranged from 20.6 to 42 points (out of 100, from worst to best).

**Conclusion:**

The use of a permanent cement spacer is a reliable solution to PSI in low-demand, older patients with comorbidities, a population in whom it is desirable to avoid additional surgery. Our review found a high rate of infection eradication and moderate-to-good objective and subjective results. However, the overall level of evidence of included studies was very low, and higher-quality studies are needed to clarify the role of permanent spacers in the treatment of PSI.

**Electronic supplementary material:**

The online version of this article (10.1007/s11420-020-09755-7) contains supplementary material, which is available to authorized users.

## Introduction

Peri-prosthetic shoulder infection (PSI) is a rare but often devastating complication of shoulder joint replacement [5, 14]. The incidence of PSI after primary shoulder arthroplasty is estimated to be 1 to 5%, depending on the study; after revision surgery, however, the incidence can reach 15% [49, 55]. Operative approaches including debridement, antibiotics, and implant retention (DAIR) and one- and two-stage revision surgery have been used over the years, but the debate over which is the best for managing this disabling condition is still open [10, 27, 28].

Two-stage and one-stage revisions have been considered the treatments of choice for PSI, although the variability in functional outcomes, the risks related to multiple surgical procedures, and the challenges in managing bone loss during reimplantation are considerable concerns [13, 27, 31, 52]. In order to overcome pitfalls associated with two-stage approaches, researchers have been studying the implantation of an antibiotic-loaded articular cement spacer as a treatment for PSI in selected low-demand patients (those not expected to engage in strenuous shoulder movement) [7, 15, 24, 40, 53]. The purpose of this systematic review of the literature was to assess clinical and functional outcomes in patients treated with a permanent spacer in the setting of a PSI, in order to define the rate of infection eradication, biomechanical reliability, and appropriate indications for this treatment.

## Search Strategy and Criteria

We used the *Cochrane Handbook of Systematic Reviews of Interventions* and Preferred Reporting Items for Systematic Reviews and Meta-Analyses (PRISMA) as criteria for conducting this systematic review of the literature [11, 34] (see Table 1). We included prospective and retrospective studies that evaluated clinical outcomes of antibiotic spacer use for the treatment of PSI in this systematic review.

**Table 1:** Characteristics of included studies

First author	Year	Mean age (SD)	No. of patients	No. free from infection	Eradication rate (%)	Active ROM	Subjective post-operative scores; mean (SD)	Surgical technique; type of spacer with antibiotics	Duration of antibiotic therapy	Comorbidities	Complications	Mean follow-up (range)	Strength(s) and weakness(es)	Level of evidence
Themistocleous [53]	2007	64	11	9	81.8	Ab 75°; ER 25°	QuickDASH 37.5	Debridement, irrigation with 10 L saline solution; custom-made spacer with vancomycin/tobramycin		3 CRD, 3 cirrhosis, 2 DM	None	22 months (15–26)	S: outcome measures, type of intervention W: bias in patient selection	IV
Coffey [7]	2010	58.9	4	4	100	El 110°^a^; ER 20°^a^	Constant 57^a^; ASES 74^a^; SST 6.6^a^; UCLA 26^a^; VAS 0.5^a^	Debridement, irrigation with 10 L saline solution; InterSpace Exatech with gentamicin			None	19.3 months (16–25)^a^	S: type of intervention W: bias in patient selection and outcome measures	IV
Stine [51]	2010	ND	15	15	100	Ab 71°; ER 29°; El 73°	DASH 50; SST 5	Debridement, irrigation 9 L saline solution, 1 L saline with polymyxin/bacitracin; custom-made spacer with vancomycin/tobramycin	6 weeks post-operatively		2 fractures	28.8 months	S: type of intervention and outcome measures W: bias in patient selection	IV
Jawa [24]	2011	ND	12	10	83.3	El 83°	ND	DePuy Prostalac® with gentamicin/tobramycin			1 dislocation, 3 fractures, 5 infection recurrences, 1 glenoid erosion, 6 revisions	27.6 months (12–69)^a^	S: type of intervention, patient selection W: outcome measures, missing data	IV
Verhelst [54]	2011	63.5 (9.6)	10	10	100	ER 21°^a^	Constant 36.3 (21); VAS 2.6^a^	Debridement, irrigation with 15 L saline solution; custom-made spacer with gentamicin	3 months post-operatively		2 peri-prosthetic fractures, 1 revision	46.4 months^a^	S: control group, patients selection, type of intervention W: outcome measures	III
Romanò [44]	2012	63^a^	15	14	93.3	Ab 51°; ER 13°	Constant 34; VAS 1.5	11 custom-made, 4 commercially produced spacers	6 weeks to 5 months		None	36 months	S: outcome measures, patient selection W: bias in type of intervention	IV
Ghijselings [15]	2013	59.4 (13.1)	5	5	100	ND	Constant 20.6 (11.3); DASH 71 (21); ASES 67; SST 1 (1.1); VAS 6 (2.3)	Prefabricated spacer with gentamicin	6 weeks clindamycin/vancomycin	2 cerebrovascular disease, 1 DM, 3 BMI > 30	1 humeral fracture	64.8 months (12–87)	S: outcome measures, type of intervention W: patient selection	IV
Levy [29]	2015	72.3	9	9	100	El 90°; Ab 70°	ASES 65.8; SANE 54.6; VAS 2	Size 6 DJO Surgical Foundation humeral hemiarthroplasty stem coated with cement with tobramycin/vancomycin and Foundation humeral head	6 months post-operatively		None	25 months (12–48)	S: type of intervention, outcome measures W: patient selection	IV
Mahure [30]	2016	73 (9)	9	9	100	El 67°	ASES 57 (24)	8 prefabricated spacers with gentamicin, 1 custom-made spacer with tobramycin		2 Parkinson’s disease, 1 DM, 3 arterial hypertension, 2 thyroid pathology	1 peri-prosthetic fracture (healed in valgus), 1 glenoid erosion	48 months	S: type of intervention, outcome measures W: patient selection	IV
Grubhofer [17]	2018	ND	14	12	92.9	ND	Constant 42 (10); SSV 29 (25); constant pain element score 11.1 (4.3)	Prefabricated spacer with gentamicin and vancomycin + bent 3.5 dynamic compression plate	2 weeks IV + 10 weeks oral post-operatively: amoxicillin + clavulanic acid + targeted IV		2 infection recurrences, 2 revisions	ND	S: patients selection, control group, type of intervention W: outcome measures, missing ROM data	III
Pellegrini [39]	2019	70.2 (10.2)	19	19	100	Ab 51.6°; El 58.2°	Constant 37.8 (17.1); VAS 1.6 (1.8)	12 preproduced, 7 custom made spacers with clindamycin/gentamicin			None	8 years (2–10)^a^	S: patients’ selection, control group, type of intervention, outcome measures W: outcome measures, missing ROM data	III
Patrick [38]	2019	63.6 (11.2)	20	17	85	Ab 52.1°; ER 3.6°; El 48.6°	Constant 33.8 (16.4); ASES 45.5 (19.3); SPADI 65.3 (18.6); SST 4.3 (3.4); UCLA 15 (4.2); SF-12 28.6 (6.3); VAS 4.5 (2.7)	Prefabricated spacer with gentamicin		5 DM, 6 immunocompromised patients, 2 CRD	2 post-operative fractures, 1 iatrogenic fracture, 1 wound healing complication, 2 hematomas, 3 infection recurrences	ND	S: patients’ selection, control group, outcome measures W: missing data, surgical technique, and follow-up.	III

*PSI* peri-operative shoulder infection, *SD* standard deviation, *ROM* range of motion, *Ab* abduction, *ER* external rotation, *QuickDASH* Quick Disabilities of the Arm, Shoulder, and Hand outcome questionnaire (range 0 to 100, no disability to severe), *CRD* chronic renal disease, *DM* diabetes mellitus, *S* strength(s), *W* weakness(es) *El* elevation, *Constant* Constant–Murley Shoulder Outcome Score (range 0 to 100, worst to best shoulder function; for pain only [Grubhofer], 0 to 15), *ASES* American Shoulder and Elbow Surgeons Standardized Shoulder Assessment Form (range 0 to 100, worse to better shoulder condition), *SST* Simple Shoulder Test (range 0 to 12, worst to best shoulder function), *UCLA* University of California Los Angeles Shoulder Score (range 0 to 35, worse to better shoulder function), *VAS* visual analog scale (range 0 to 10, no pain to worst pain), *ND* no data, *DASH* Disabilities of the Arm, Shoulder, and Hand outcome questionnaire (range 0 to 100, no disability to most severe disability), *BMI* body mass index, *SANE* Single Assessment Numeric Evaluation (range 0 to 100%, 100% being normal), *SSV* Subjective Shoulder Value (range 0 to 100%, 100% being normal), *IV* intravenous, *SPADI* Shoulder Pain And Disability Index (range 0 to 130, 0 less shoulder disability and 100 more shoulder dysfunction), *SF-12* 12-Item Short Form Health Survey (range 0 to 100, lowest to highest level of health)

^a^Data referred to the overall study population

Studies that evaluated the use of a permanent antibiotic-loaded spacer in revision surgery for PSI were considered eligible for the systematic review. Eligible studies also did not place age restrictions on their pool of participants. Criteria used to confirm PSI were the Musculoskeletal Infection Society criteria [20, 37], synovial fluid analysis (with a white blood cell count over 3000 mm^3^ and polymorphonuclear neutrophil proportion greater than 80 as thresholds), and deep biopsy of peri-prosthetic tissues. Devices included for review were preformed or prefabricated spacers, custom-made cement spacers, and cement-coated hemiarthroplasty stems.

The infection-eradication rate was the primary outcome measure. Secondary outcome measures were active range of motion (ROM), clinical scores on shoulder function tests, level of pain, and post-operative complications. Studies reporting only results of other treatments (one- or two-stage revisions, resection arthroplasty, or infection-suppression therapy) and case reports were excluded, as were original studies conducted using animal models or cadaveric specimens, studies lacking abstracts or quantitative details, editorial commentary, technical notes, and non-English language papers.

We conducted a systematic search of the literature published between January 1, 1980, and September 1, 2019, in the following databases: the Cochrane Central Register of Controlled Trials (CENTRAL), MEDLINE/PubMed, Embase, Scopus, the Science Citation Index Expanded from Web of Science, ScienceDirect, CINAHL, and LILACS. In the search, we used various combinations of the following keywords: “shoulder infection,” “arthroplasty,” “one stage,” “two stage,” “periprosthetic joint infection,” and “spacer.” Only original studies published in English in peer-reviewed journals were considered.

Two independent reviewers (A.R. and A.C.) screened each title and abstract retrieved in the electronic searches. Full text versions of studies were retrieved when the title or abstract referred to PSI treatment. The reviewers followed an identical checklist to screen studies before including them for analysis. The references of all included articles were screened for papers that had been missed in the database search.

Data analysis was performed in duplicate, and disagreements were resolved through consultation with a third reviewer (M.A.M.). When studies did not provide complete data, the authors were contacted if possible. Mean values of data presented in results were obtained exclusively from subgroups treated. The overall quality of studies included in this review was assessed using the Grading of Recommendations, Assessment, Development and Evaluations (GRADE) system [19].

The risk of bias in each study included in this survey and the overall risk of bias of our review were evaluated and classified using the Risk of Bias in Non-randomized Studies—of Interventions (ROBINS-I) tool [50].

## Results

**Fig. 1:**
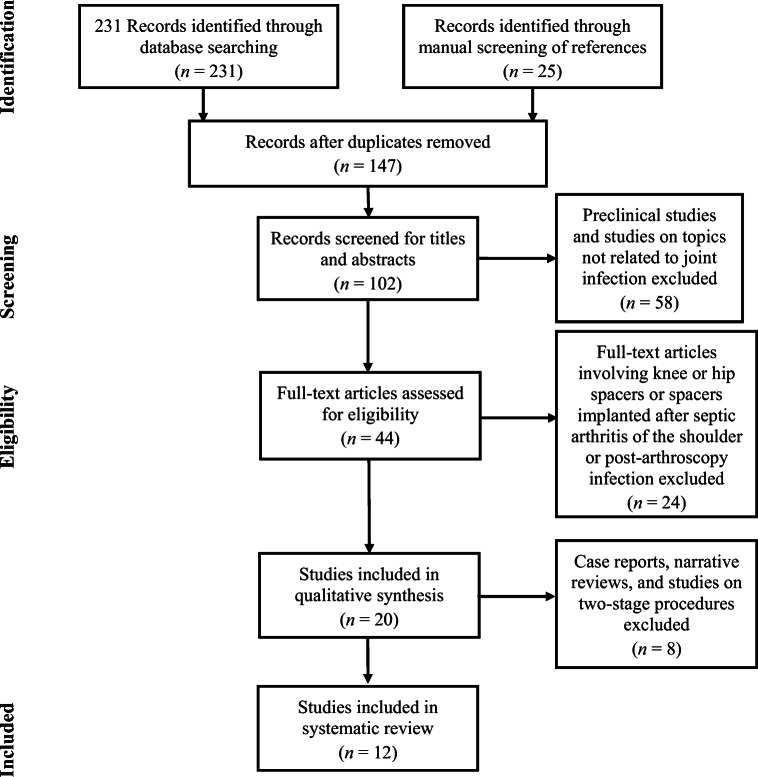
Preferred Reporting Items for Systematic Review and Meta-analysis (PRISMA) diagram.

We identified a total of 256 studies for initial evaluation. After preliminary screening and exclusion of duplicates, 102 studies were available for title and abstract screening. Fifty-eight records were excluded because they were preclinical studies or not related to joint infection. After full-text evaluation, 24 papers were excluded because they involved knee or hip spacers or permanent spacers implanted after primary septic arthritis or post-arthroscopy infection. Eight papers were excluded because they were case reports or narrative reviews or because they reported data on two-stage procedures (Fig. 1). A total of 12 articles were designated for final evaluation and systematically screened. Four studies were classified as level III evidence; the remaining eight studies were level IV. A summary of the 12 studies, including details about mean active ROM, results of subjective evaluation forms, surgical technique, and complications registered at the latest follow-up, is shown in Table 1.

All of the included studies were retrospective, and according to the Grading of Recommendations Assessment, Development and Evaluation (GRADE) system, the overall quality of evidence was low. A lack of standardization among papers in diagnostic criteria and assessment of clinical outcomes was observed. According to the ROBINS-I tool, there were serious risks of bias as a result of unmeasured confounding domains, selection bias related to both interventions and outcomes, missing data, and small numbers of participants.

A total of 143 patients treated with a permanent spacer in the setting of a PSI were included. The mean age was 65.6 years (range, 58.9 to 73 years), and the mean follow-up was 37.4 months (range, 22 to 64.8 months). Only four studies (33.3%) presented data on comorbidities of patients treated with permanent spacers; diabetes mellitus, chronic renal disease, and immune system pathologies were frequently observed.

All 12 studies described the surgical technique and type of spacer involved in detail. Six (50%) provided detailed information on antibiotic therapy protocols (see Table 1 for details on the studies). In 79 cases (55.2%), a commercially produced spacer was used. In 55 cases (38.5%), a custom-made cement spacer was used; of these, 14 (9.8% of 143) of the cement spacers were reinforced with a bent dynamic compression plate. In nine cases (6.3%), the functional spacers were made with cement-coated size 6 hemiarthroplasty stems and metal humeral heads. All of the spacers were antibiotic loaded one or more of the following: tobramycin, gentamicin, vancomycin, and clindamycin.

Four studies (33.3%) reported details about intra-operative irrigation, which was carried out with 9 to 16 L of saline solution. In one study, additional irrigation with saline solution mixed with polymyxin and bacitracin was performed. Six studies (50%) provided information about post-operative antibiotic regimen, with therapy durations ranging from 6 weeks to 6 months. The number of participants was insufficient for a meaningful subgroup analysis of individual surgical techniques.

All 143 patients were evaluated post-operatively for reinfection, functioning of the affected shoulder, and complications. Results of active ROM testing of the operated shoulder or scores obtained from subjective forms were recorded. Of the 12 studies analyzed, ten (83.3%) reported on active ROM expressed as degrees. Eleven (91.7%) presented quantitative data on patient satisfaction extracted from at least one of the following subjective functional assessments: the American Shoulder and Elbow Surgeons Standardized Shoulder Assessment Form (ASES), the Constant–Murley Shoulder Outcome Score (Constant–Murley score), the Disabilities of the Arm, Shoulder, and Hand (DASH) outcome questionnaire, the Quick Disabilities of the Arm, Shoulder, and Hand (QuickDASH), the Shoulder Pain And Disability Index, the Simple Shoulder Test, the Single Assessment Numeric Evaluation, the Subjective Shoulder Value, the 12-Item Short Form Health Survey, the University of California Los Angeles Shoulder Score, and the visual analog scale (VAS) [3, 8, 18, 21, 33, 43, 45].

Of the 143 patients included in this review, 133 (93.0%) were considered free from infection at the latest follow-up.

Functional ROM was achieved in most patients with acceptable objective results*.* The mean post-operative active elevation of the shoulder ranged from 48.6 to 90°, abduction from 51 to 75°, and external rotation from 3.6 to 29°.

The functional and clinical assessments showed moderate-to-good results in terms of post-operative active ROM, subjective scores, pain, and overall patient satisfaction. The Constant–Murley score was calculated in seven (58.3%) studies, with mean values ranging from 20.6 (unsatisfactory) to 42 (good). The ASES score was obtained in four (33.3%) of the studies, with mean values ranging from 57 to 67 (good). The DASH questionnaire scores were obtained in three studies (25%), with mean values ranging from 37.5 (good) to 71 (fair). The post-operative VAS for pain was administered in seven studies (58.3%), with mean values ranging from 1.5 (mild pain) to 6 (moderate pain) on a scale of 0 to 10.

Complications were specifically mentioned in all the included studies. The most common complication was recurrence of infection, which was described in ten patients (7.0%). Fracture of the antibiotic-loaded cement implant was reported in eight cases (5.6%). Peri-prosthetic fractures were observed in three (2.1%) cases. Only one case (0.1%) of cement-spacer dislocation was described.

## Discussion

This systematic review of the literature summarizes the latest evidence on the treatment of PSI with permanent antibiotic-loaded cement spacers, as it relates to reliability of treatment, eradication rates, functional outcomes, and indications for this surgical option. The literature suggests that permanent cement spacers represent a reliable solution for low-demand older patients with comorbidities who are unfit for further surgery. The studies revealed a high mean eradication rate (93.0%), few complications, and moderate-to-good functional outcomes.

It is important to highlight that considerable limitations affect this systematic review. First, the overall level of evidence of the included studies was low, according to the GRADE evaluation system [19]. The majority of the studies were retrospective in design, and in only a few cases were clear statistical analysis of subgroups and quantitative assessment of clinical data presented. A second limitation is the heterogenous selection of spacers included in the analysis (preformed or prefabricated spacers, custom-made spacers, and cement-coated hemiarthroplasty stems). Moreover, the functional antibiotic spacers described by Levy [29] are not true cement spacers but cement-coated hemiarthroplasty spacers. These implants have been used in a very limited population, precluding strong conclusions regarding the devices’ safety and effectiveness. Certainly, these metal implants are expected to have better mechanical properties than true cement spacers, but the reported results are in alignment with those reported by other authors. No clinical trials were included in our review. Standardization in reported diagnostic, surgical, and post-operative assessment protocols was also lacking and severely limited our ability to perform comprehensive quantitative analysis of clinical outcomes. Therefore, concerns over the risk of bias, as it relates to the ROBINS-I assessment, affect our results [50].

Management of a large number of conditions including osteoarthritis, avascular necrosis, and fractures requires shoulder arthroplasty to restore function and relieve pain. As has been observed with hip and knee arthroplasty, the growing number of shoulder arthroplasties being performed leads to a rising incidence of PSI [4, 5]. Reverse shoulder arthroplasty seems to be associated with a higher risk of infection than anatomic total shoulder arthroplasty, and the risk is even higher in the setting of revision surgery [23, 36, 41, 48]. Several approaches have been investigated for the diagnosis and management of PSI, but the clinical implications of the condition are still challenging for orthopedic surgeons. DAIR is a less invasive procedure that involves debridement, accurate irrigation, the exchange of all mobile components, and antibiotic therapy in an effort to retain the prosthetic implant. This technique is indicated only in cases of acute PSI and has yielded uncertain results in terms of infection eradication [9, 12, 44].

Two-stage revision is the treatment of choice for chronic PSI in younger and high-demand patients, even if the management of bone loss can be challenging [35, 46, 52]. Satisfactory results of one-stage revision have been seen in cases of an identified causative pathogen, allowing for lower costs and lower risk, as compared with multiple surgical procedures [2, 10, 16, 22, 26]. However, a recent systematic review and meta-analysis by Kunutsor et al. [27] suggested that there is no statistically significant difference in reinfection rate between one- and two-stage procedures.

Resection arthroplasty is a salvage procedure and is associated with poor functional results and pain control [42, 47]. For this reason, the technique should be reserved only for cases of recalcitrant PSI with extensive bone loss in low-demand and older patients [6]. Despite reports of poor clinical results [6, 38, 43], Verhelst et al. [54] demonstrated that preservation of tuberosities could play a role in improving functional outcomes of resection arthroplasty.

Use of a permanent antibiotic-loaded cement spacer has been proposed as a definitive treatment for PSI [25, 40, 53]. Antibiotic-loaded cement spacers are commonly used as part of two-stage procedures in order to control infection and maintain adequate limb length during the inter-stage period [39]. Theoretically, the spacers release antibiotic molecules into the surrounding tissue for a limited period with predictable pharmacokinetics [1], restoring function, improving tensile strength throughout the peri-articular soft tissue, and relieving pain [29, 30]. Moreover, the possibility of customizing cement-spacer implants could be advantageous in managing bone loss and improving function [44, 51, 53, 54]. However, some weaknesses related to the mechanical properties of spacers still persist, and several cases of failure have been reported in the literature [17, 32, 38]. In the current review, infection recurrence was the most commonly observed complication, followed by peri-prosthetic fracture and dislocation. Some studies included in the review compared a group of patients treated with a permanent spacer with patients who underwent two-stage revision [17, 38, 39]; in these studies, no differences in eradication of infection or complication rates were observed, although better ROM results were still observed among patients who underwent two-stage reimplantation. Ten (83.3%) of the 12 studies analyzed quantitative data on post-operative ROM, and in all cases, the functional results and subjective satisfaction scores were considered acceptable. Patrick et al. showed significantly better results in the Constant–Murley score and ROM in patients treated with two-stage revision, in comparison with a cement-spacer group, although it is interesting that no differences in subjective satisfaction scores or VAS scores were seen [38].

The most important finding of this systematic review is the high infection-eradication rate (93.0%) with the use of a cement spacer. Furthermore, shoulder spacers showed good mechanical reliability over time. Given the low rates of infection recurrence and mechanical complications and the good functional results that emerged from this review, the use of a cement spacer should be considered as a definitive treatment of PSI in older and low-demand patients at high risk for complications related to chronic comorbidities—in other words, patients in whom further surgery should be avoided.

In conclusion, although one- and two-stage revisions remain the treatments of choice for PSI, the use of a permanent antibiotic-loaded cement spacer is a reliable solution in low-demand older patients with comorbidities who are unfit for further surgery. The studies included in the review showed high rates of infection eradication, as well as moderate-to-good results in terms of post-operative active ROM, subjective clinical outcomes, and overall patients’ satisfaction. The most common complications were infection recurrence and spacer and peri-prosthetic fractures. However, the level of evidence of the studies included in the systematic analysis was low and presented high risks of bias; high-quality, multicenter, prospective studies are needed to clarify the role of permanent spacers in eradicating PSI and restoring shoulder function.

## Electronic supplementary material

ESM 1 (PDF 1224 kb)

ESM 2 (PDF 1224 kb)

ESM 3 (PDF 1224 kb)

ESM 4 (PDF 1224 kb)

ESM 5 (PDF 1224 kb)

ESM 6 (PDF 1224 kb)

ESM 7 (PDF 1224 kb)

ESM 8 (PDF 1224 kb)
